# Longitudinal Influences of DRD4 Polymorphism and Early Maternal Caregiving on Personality Development and Problem Behavior in Middle Childhood and Adolescence

**DOI:** 10.3389/fnhum.2022.839340

**Published:** 2022-04-14

**Authors:** Peter Zimmermann, Gottfried Spangler

**Affiliations:** ^1^Institute of Psychology, Department of Developmental Psychology, University of Wuppertal, Wuppertal, Germany; ^2^Institute of Psychology, Friedrich-Alexander-Universität Erlangen-Nürnberg, Erlangen, Germany

**Keywords:** DRD4, dopamine, maternal sensitivity, personality development, gene-environment (GxE) interaction, ego-resiliency, ego-undercontrol, aggressiveness

## Abstract

Most studies examining gene-environment effects on self-regulation focus on outcomes early childhood or adulthood. However, only a few studies investigate longitudinal effects during middle childhood and adolescence and compare two domains of early caregiving. In a longitudinal follow-up with a sample of *N* = 87, we studied the effects of differences in the DRD4 tandem repeat polymorphisms and two domains of early maternal caregiving quality on children’s personality development using Block’s California Child Q-Set (CCQ) at age six and age 12 and on problem behavior at ages six and seven. Early maternal regulation quality predicted later ego-resiliency and aggressiveness. In addition, significant gene-environment interactions revealed that children with the 7+ DRD4 tandem repeat polymorphism and poor maternal regulation quality in infancy showed lower scores in ego-resiliency and higher scores in ego-undercontrol and CCQ aggressiveness. In contrast, children who had experienced effective maternal regulation in infancy showed a comparable level in personality traits and problem behavior as the DRD4 7- group independent of the levels of maternal regulatory behavior. Similarly, longitudinal caregiving × DRD4 interactions were found for behavior problems in middle childhood, especially for oppositional-aggression, inattentive-hyperactivity, and social competence. Early caregiving effects were only found for maternal regulation quality, but not for maternal responsiveness. Effective early maternal regulation in infancy can moderate the negative effect of DRD4 7+ on children’s self-regulation in middle childhood and adolescence. However, maternal responsiveness has no comparable effects. It seems relevant to consider several dimensions of early caregiving and to also measure the environment in more detail in gene-environment studies.

## Introduction

Although attachment theory emphasizes the importance of caregivers’ emotional availability and appropriate support for child development, Bowlby’s concept of the development of emotionally stable personality traits was an interaction between genetic dispositions and caregiving experiences (Bowlby, 1973). He included Waddington’s epigenetic model (Waddington, 1942) in attachment theory and suggested that an emotionally stable person develops as the outcome of continuing genetic and supportive environmental interactions. In a transactional perspective (Sameroff, 2010), differences in personality characteristics (e.g., emotional stability, self-regulation,) consequently influence the probability of individual adjustment or maladjustment when facing later adversities or challenges during the life course (Bowlby, 1988).

### Attachment, Caregiving, and Personality Characteristics

Research on social development and specifically attachment development has repeatedly demonstrated that secure attachment and supporting parenting are associated with personality characteristics that assess emotional stability and socially responsible behavior. Ego-control and ego-resiliency (Block and Block, 2006) are such personality traits. Ego-control describes the habitual tendency to control one’s impulses and emotions, ranging from poor delay of gratification and impulsiveness even when inappropriate (ego-undercontrol) to inhibition even when not necessary in that specific situation (ego-overcontrol). Ego-resiliency describes the situation-appropriate modulation of ego-control (Block and Block, 2006), i.e., the ability to adapt the level of ego-control to the requirements of a specific context or situation. Both personality characteristics are relatively stable over time, already starting in early childhood (Block and Block, 2006; Chuang et al., 2006; Taylor et al., 2014). Studies repeatedly showed associations of both ego-resiliency and ego-undercontrol with concurrent and later adaptation, psychopathology, and social or academic success (Rothbaum and Weisz, 1994; Robins et al., 1996; van Aken et al., 2002; Martel et al., 2007; Syed et al., 2020).

Secure attachment in infancy, middle childhood, and adolescence is associated with higher levels of ego-resiliency and moderate levels of ego-control (Arend et al., 1979; Kobak and Sceery, 1988; Suess et al., 1992; Zimmermann et al., 1996; Zimmermann, 1999; Kersten-Alvarez et al., 2010; Caldwell and Shaver, 2012; Zimmermann and Scheuerer-Englisch, 2013). Infant attachment security is also longitudinally associated with low neuroticism, high agreeableness, and conscientiousness in adulthood (Young et al., 2019), specifically those Big Five domains that also characterize ego-resilient individuals (Scholte et al., 2005).

Moreover, also caregiving sensitivity is associated with ego-resiliency. Stams et al. (2001) reported that higher caregiver sensitivity towards infants as a result of an experimental intervention was longitudinally associated with higher ego-resiliency at age 7 (for girls), whereas maltreatment or parental intrusiveness was associated with lower ego-resiliency (Kim et al., 2009; Taylor et al., 2013). Moreover, support of children’s emotional needs and supportive guidance during exploration and problem-solving (Block et al., 1998; Kremen and Block, 1998) as well as early child-care (Wessels et al., 1997) are associated with higher ego-resiliency scores. Thus, there is some empirical evidence for concurrent and longitudinal effects of caregiving and attachment security on the development of personality characteristics that specifically assess adaptive self-regulation (e.g., ego-resiliency).

### Effects of Gene × Environment Interaction on Personality Development

Despite the many studies that examine gene × environment interactions on signs of dysregulation in developmental psychopathology (Moffitt et al., 2006; Thapar et al., 2007a; Manuck and McCaffery, 2014; Pinto et al., 2015; King et al., 2016) or effortful control in childhood and adolescence (Cho et al., 2016; Ganiban et al., 2021) only few studies examined gene × environment interaction on ego-resiliency development. Taylor et al. (2014) examined the longitudinal influences of early maternal caregiving behavior and two variants of the serotonin transporter polymorphism on the development of ego-resiliency from toddlerhood to middle childhood. They reported two main effects. Early sensitivity at the age of 18 months was associated with concurrent ego-resiliency (but not with ego-resiliency in middle childhood) and in addition, the haplotype of the two variants of the serotonin transporter polymorphism (5-httlpr and SERT intron 2) was associated with higher ego-resiliency scores in early childhood, but again not with ego-resiliency during middle childhood. However, they did not find evidence for a gene × environment interaction using a composite score of maternal sensitivity, warmth, intrusiveness, and control as caregiving index.

In contrast, studies focusing on genetic polymorphisms affecting the functionality of the dopamine system, specifically the DRD4 polymorphism, report direct effects as well as the moderation of genetic effects by environmental factors on developmental outcomes in the domain of self-regulation, impulsivity, and externalizing behavior. The DRD4 gene is one of those candidate genes that repeatedly but not consistently is associated with specific aspects of temperament and personality (Savitz and Ramesar, 2004). Associations between a variant of the dopamine D4 receptor gene (the 7-repeat allele of the 48 base pair repeat sequence; DRD4 7+) have been found already with infant temperament, sensation seeking, and attention deficit disorder (Ebstein et al., 1998, 2000; Lakatos et al., 2003; Faraone et al., 2005; Birkas et al., 2006). The DRD4 gene is functionally associated with the signal transmission of the dopamine system regulating many executive functions like control and inhibition of attention and action. There is ample evidence that the DRD4 7+ variant is associated with increased difficulties in self-control, executive functioning, or signs of ADHD in children (Schmidt et al., 2001; DiLalla et al., 2009; Pappa et al., 2015), but also for DRD4 × environment interaction effects (Martel et al., 2011). King et al. (2016) reported that infants carrying the DRD4 7+ allele showed increased rates of externalizing behavior when mothers showed low sensitivity. Other studies report similar interaction effects between DRD4 polymorphism status and low maternal sensitivity on later externalizing problems and ADHD for older children and adolescents (Bakermans-Kranenburg and van IJzendoorn, 2006; Nikitopoulos et al., 2014). In a detailed analysis, Elam and DiLalla (2018) showed that, even during a short mother-child interaction of 10 min, children carrying the DRD4 7+ allele became less responsive towards their mothers and mothers became less sensitive. The interaction effect of maternal responsiveness and DRD4 7+ on the CBCL dysregulation profile in middle childhood was only obvious when infants additionally showed early regulatory problems (Poustka et al., 2015). Moreover, as shown in the large NICHD-study, young children who are carriers of the DRD4 7+ variant showed less delay of gratification and more inattention or impulsivity when experiencing more hours of daycare compared to DRD4 7- carriers (Berry et al., 2013). However, Propper et al. (2007) also reported that African American children with the short polymorphism of the DRD4 showed less externalizing behavior when their mothers showed more warmth. They emphasize the relevance of different effects of specific parenting behaviors. Specifically, maltreatment may play a crucial role. Thibodeau et al. (2015) reported that children with specific genetic variations of dopaminergic genes showed a higher environmental sensitivity for the development of impulsivity (i.e., ego-undercontrol) and consequently developed more anti-social behavior when experiencing maltreatment. Therefore, it seems reasonable to expect effects of genetic differences that affect the dopamine system on those personality characteristics that directly assess behavior tendencies of self-control as well as capacities for self-regulation and modulation of self-control, as described by Block and Block (1980) with their concept of ego-control and ego-resiliency. Moreover, also other individual differences in self-regulation (e.g., aggression, attention problems) might well be influenced by variants of the DRD4 gene and moderated by specific aspects of caregiving.

### Caregiving and Self-Regulation: Timing and Domain-Specific Effects

Research on the association between caregiving and children’s self-regulation has shown mixed results (Karreman et al., 2006; Bridgett et al., 2015). The timing of caregiving experiences (i.e., early childhood vs. late childhood) and differences in specific caregiving behaviors can play a decisive role in the development of self-regulation and related behavior problems.

The relevance of timing in caregiving has become obvious in the EARA study on differential effects of early deprivation and later adoption (O’Connor et al., 2003). From childhood until early adulthood, the later adoption group (after the age of 6 months) showed increased and enduring attentional problems compared to the early adoption group (Sonuga-Barke et al., 2017). Early deprivation or early maltreatment has long-lasting effects on self-regulation at the physiological level (Gunnar and Pollak, 2007; Gunnar and Quevedo, 2007) and at the level of information processing (Pollak and Sinha, 2002; Wismer Fries et al., 2005). These studies suggest the importance of early caregiving experiences for self-regulatory processes in the area of attention, social behavior, emotional and social cognition. Similarly, early intervention programs focusing on changing the parent-child interaction seem to be more effective in influencing self-regulation or executive functions compared to later interventions (Hentges et al., 2020).

The relevance of a specific parenting domain is another source contributing to the differences in research results on the association between caregiving and self-regulation (Belsky, 1984). In a meta-analysis, Karreman et al. (2006) only found small effect sizes for the general association between parenting and self-regulation in childhood. Moreover, Grusec and Davidov (2010) have pointed out that different parenting domains have different outcomes in child development. In their review of the socialization literature, maternal warmth was associated with children’s felt security but not necessarily with their self-regulation. We, therefore, assume that specifically experiences of effective emotional regulation, autonomy support, and limit-setting contribute to the development of self-regulation (Kochanska et al., 2000; Bernier et al., 2010).

Differential effects of specific parental behaviors can also be found in the case of maternal sensitivity which includes the processes of perception, correct interpretation, prompt reaction, and regulation of the infant’s emotions or needs (Ainsworth, 1967). Although these components are interrelated, especially the regulation of the infant’s needs is predictive of a low rate of crying, whereas the perception or prompt reaction (i.e., responsiveness) is not (Lohaus et al., 2004).

The role of specific aspects of sensitive caregiving on self-regulation is also obvious in gene × environment studies. Sheese et al. (2007) showed that high parenting quality in infancy moderates the effect of the DRD4 repeat allele (7+) on sensation seeking (i.e., activity, high pleasure, impulsivity), however not on effortful control (of attention). Similarly, maternal responsiveness but not maternal effective regulation moderated the effect of 5-httlpr polymorphism on attachment disorganization (Spangler et al., 2009).

Thus, the experience of external regulation, but not the fast caregiver reaction is a predictor of a child’s effective self-regulation. This is especially important in infancy when individuals rely on external emotion regulation provided by the parents (Thompson, 1993). The effective regulation of the infant’s attachment needs and also of the increasingly salient need for autonomy and exploration are of special importance for the development of self-regulation.

### Aims of the Study

Given these previous findings, the present study aimed at examining the interplay between early social caregiving experiences and allelic variations of the DRD4 gene in the longitudinal development of self-regulatory competencies. We tested this specifically for: (1) ego-resiliency and ego-control as personality characteristics assessing self-regulation, (2) aggressiveness and anxiety as personality characteristics that represent dysregulation but not yet clinical symptoms, and (3) problem behavior as clinical indicators of enduring dysregulation.

Following the hypothesis that maternal regulatory behavior and not only maternal responsiveness to the infant’s signals is influential for the development of self-regulation, we separately examined the effect of the two maternal caregiving variables: (1) maternal regulation of the infant’s emotions and (2) maternal responsiveness to the infant’s needs.

From a developmental perspective, we additionally wanted to test whether the effects of gene × environment interaction or personality development are depending on age. Savitz and Ramesar (2004) conclude that effects of the DRD4 polymorphism are more prominent at a younger age. In addition, Reiss and Leve (2007) emphasize that the activation of genes might be age-specific and that early genetic effects might influence later gene-environment interaction. Moreover, Schmidt et al. (2001) showed DRD4 effects on the stability of attention problems. Thus, the analysis of age effects was included. Finally, we also examined possible effects of gene × environment correlations or evocative processes (Moffitt et al., 2006; Taylor and Kim-Cohen, 2007).

In sum, we had three main aims:

(1) to study gene-environment interaction of DRD4 polymorphisms and early caregiving on the longitudinal development of self-regulation capacities, (a) at the level of personality characteristics for self-regulation at age six and age 12 (ego-resiliency and ego-control), (b) individual differences in dysregulation at age six and age 12 (aggression and anxiety), and (c) behavior problems at age six and seven,

(2) to examine whether maternal regulatory caregiving predicted children’s self-regulation better compared to maternal responsiveness, and

(3) to examine age differences in these effects on personality.

## Method

### Participants and Procedure

The original sample consists of 106 healthy German, Caucasian, low-risk infants (53 girls/53 boys) and their mothers stemming from a wide range of socioeconomic statuses. Families included in the study at the time of recruitment were all two-parent families, only children with gestation age between 38 and 42 months, no pre-term birth, no handicaps or severe illnesses, or long hospitalization during the first year of life. The mothers were the infant’s primary caretakers in all families except one.We obtained informed consent from the parents at each assessment period. For more details, see Spangler and Schieche (1998).

At the age of 12 months, we observed mother-infant interaction during a 30-min competing demands free play session. Mothers answered a questionnaire while feeling free to respond to the infant, as they usually would do. In a follow-up assessment at age six with 97 subjects, mothers were asked to describe their children’s personality by means of the California Child Q-sort (CCQ) and their children’s problem behavior at age six and again at age seven by a standardized questionnaire (VBV-EL). In a follow-up assessment at age 12, we collected cheek cells from 95 of the original 106 children and from 96 of their mothers for genetic analyses. In addition, mothers again provided a description of the children’s personality using the CCQ for 95 children. For the longitudinal gene-environment analysis 87 subjects had complete data.

### Data Analyses and Measures

#### Maternal Behavior at 12 Months

The quality of maternal behavior was analyzed from the videotaped free play sessions. In an event-sampling approach, the mothers’ perceptions of the infant’s signals, as well as the promptness and appropriateness of the responses to these signals as components of maternal sensitivity, were coded. Infant signals were defined as any instance of vocalization, negative facial expression, and behavior directed to the mother (e.g., approaching, looking at mother for at least 3 s, offering an object, grasping for her questionnaire). Maternal behavior was coded regarding the two dimensions *maternal responsiveness* and *maternal regulation quality* with three single variables for each maternal caregiving dimension. *Maternal responsiveness* was assessed based on: (1) the proportion of maternal reactions to the infant’s signals (e.g., signals followed by any maternal responses, ranging from short glances or behavioral breaks indicating attention to obvious infant-directed behavior), (2) the proportion of responded signals followed by an infant-directed active behavior going beyond short glances or looks, and (3) the proportion of prompt responses (within 3 s). *Maternal regulation quality* was assessed based on (4) the proportion of appropriate responses (agreeing with the child’s wish or need, e.g., mother providing a wanted object or comforting the infant when distressed), (5) the proportion of emotionally positive responses (characterized by affectionate, respectful, and sensitive behavior), and (6) proportion of episodes with sustained regulation (mother finally comforting the infant; mother allowing the infant to play with a pencil as long as he/she wants).

Reliability was examined over 12 play situations. The rater agreement for the detection of infant signals was 81%. Kappa scores for the single maternal behavior categories ranged between 0.76 and 1.0. The three variables for *maternal responsiveness* as well as the three variables for *maternal regulation quality* showed high within domain correlations (ranging from 0.81 to 0.92). However, correlations between the two maternal caregiving domains were lower (ranging from 0.36 to 0.62). We computed separate composite scores based on z-transformed scores for maternal responsiveness and for maternal regulation quality, respectively.

*Maternal responsiveness* represents the mean proportion of the infant’s signals that the mother perceived and reacted to promptly, while *maternal regulation quality* represents the mean proportion of appropriate, emotionally positive, and sustained regulation of the infant. For statistical analysis, both maternal caregiving domains were dichotomized by the median split.

#### Children’s Personality

The California Child-Q-sort (CCQ; Block and Block, 1980) was used to assess the children’s personality traits. At age six, we used the German 54-item short version (Göttert and Asendorpf, 1989) of the original 100-items California Child-Q-sort (CCQ; Block and Block, 1980) to reduce the participant’s workload.The correlations between each child’s Q-sort and the prototypes for ego-resiliency and ego-under control (self-regulation personality characteristics; provided by Block and Block, 1980), as well as aggressiveness and anxiety (dysregulatory personality characteristics; Zimmermann et al., 2009; Zimmermann and Scheuerer-Englisch, 2013) are scores for the prototypicalities of each child for each of these dimensions. Ego-control describes the degree of habitual control of impulses and emotions ranging from a low delay of gratification and impulsivity to enduring inhibition. Ego-resiliency describes the ability to modulate the level of control appropriately depending on the situation. Aggressiveness assesses the salience of attacking others directly or indirectly, and anxiety the salience of fear and withdrawal within the personality profile of the child. The prototypes for aggressiveness and anxiety were composites of Q-sorts provided by clinical and developmental psychologists with a reliability of *r* = 0.87, and *r* = 0.83, respectively.

At age 12 we used the original 100-item German version of the CCQ as the standard procedure and as the short CCQ version had only been validated for preschool children. For the specific longitudinal analysis and in order to control for possible methodological differences between the original long and the short version, we only used the 54 items of the short version for the calculation of the prototypicalities at age 12. However, correlations between the prototypicalities of the short and the long version of the CCQ at age 12 were *r* = 0.98, 0.89, 0.97, and 0.94 for ego-resiliency, ego-undercontrol, aggressiveness, and anxiety, respectively.

#### Children’s Problem Behavior

Mothers rated their children’s problem behavior during home visits when children were six and seven years old using the VBV-EL (“Verhaltensbeobachtungsbogen für Vorschulkinder”; Döpfner et al., 1993). The VBV-EL is a 53-item standardized German checklist to assess externalizing and internalizing problem behavior and personal resources during the preschool period. It consists of four subscales, including social-emotional competence, oppositional-aggressive behavior, attention-deficit/hyperactivity, and affective problems. The VBV-EL has been applied to clinical and non-clinical samples (Sarimski, 1997; Laucht et al., 2000) and has shown high internal consistencies (alpha = 0.71–0.92), and good test-retest reliability (range *r* = 0.48–0.78) in clinical samples (Renner et al., 2004). In the present sample, the one-year stabilities of the VBV-EL subscales were good (ranging from *r* = 0.61 to 0.69). In order to ensure high reliability, we used the mean scale scores of the assessments at age six and seven for further statistical analysis.

#### Molecular-Genetic Analyses

Genotyping for children and mothers was performed at the Institute of Psychiatry, University of Regensburg (Germany) for the DRD4 exon III repeat polymorphism. Genomic DNA was isolated from buccal swabs using published procedures (Freeman et al., 1997).

For exon III 48-bp VNTR polymorphism in DRD4 primers were 5’ GCG ACT ACG TGG TCT ACT CG 3’ and 5’ AGG ACC CTC ATG GCC TTG 3’. PCR cycling conditions were 15 min for 95°C followed by 35 cycles of 45 s at 95°C (denaturation), 30 s at 50°C (annealing), and 30 s at 72°C (elongation) with a final extension for 7 min at 72°C using a Multicycler PTC 200 gradient machine (Biozym Diagnostik, Germany). PCR products were separated by 2.0% agarose gel electrophoresis and stained with ethidium bromide for UV visualization (Schoots and van Tol, 2003). For the purpose of present analyses and based on previous findings regarding the specific role of the 7-repeat polymorphism, a dichotomous measure of the DRD4 polymorphism was defined. Children were grouped in one group who at least had one 7-repeat allele (DRD4 7+) and another group who did not have any 7-repeat allele (DRD4 7-).

The allele-wise distribution of the children’s DRD4 polymorphism was comparable to European and Middle East populations (Chang et al., 1996). While the most frequent variant was the 4-repeat (67.9%), the frequency of the 7-repeat was 13.2%, and of the 2-repeat was 9.5%. The remaining rare alleles summed up to a frequency of 9.6%. Due to small cell counts for specific DRD4 genotypes, Hardy-Weinberg equilibrium was tested only for combinations of presence and absence of the 7-repeat polymorphisms, which were in the equilibrium, *χ^2^* (2, *N* = 96) = 0.32, *p* = 0.85. There was no significant effect of infant sex on the distribution of the DRD4 polymorphisms.

### Examining Gene-Environment Interactions

We examined the longitudinal effects of genetic dispositions (DRD4 (7+ vs. 7-) and early caregiving experiences (maternal regulation quality, maternal responsiveness, high vs. low, respectively) on the development of personality and problem behavior. We, therefore, categorized the child variables into three variable groups: self-regulation personality characteristics (ego-resiliency, ego undercontrol), dysregulatory personality measures (aggressiveness, anxiety), and problem behavior (oppositional-aggression, inattentiveness/hyperactivity, emotional problems, and social competence). For each of the groups, a multivariate analysis of variance was conducted with DRD4 (7- vs. 7+) and maternal regulation (low vs. high) as independent factors. For the personality measures, we used age (6 vs. 12 years) as a repeated measures factor in addition. We similarly conducted a MANOVA using maternal responsiveness instead of maternal regulation. In the case of significant overall effects, separate univariate MANOVAs were conducted. To disentangle the interaction effects between DRD4 and maternal behavior, separate maternal regulation × age MANOVAs were conducted for the two DRD4 groups, and separate DRD4 × age MANOVAs were conducted for the two maternal behavior groups, as *post-hoc* tests.

## Results

### Preliminary Analyses

We first report the distribution of the children’s DRD4 polymorphisms and examine potential associations of maternal caregiving behavior with their own or their children’s variations of the DRD4 polymorphism.

Twenty-three of the 87 children (26%) with a complete data set possessed at least one DRD4 7-repeat polymorphism (7+), while 74% did not (7-). This is comparable to the total sample, where 24 (25%) of the 95 children showed the DRD4 7+ polymorphism. A MANOVA showed that maternal regulation quality and maternal responsiveness did not differ as a function of children’s DRD4 status (7+ vs. 7-; *F*_(2,91)_ = 0.72, *p* = 0.489), indicating that maternal caregiving was not influenced by the DRD4 polymorphisms status of the child. Similarly, maternal caregiving was not related to her own DRD4 polymorphism status (*F*_(2,92)_ = 0.19, *p* = 0.828). Thus, differences in mothers’ responsiveness and regulation quality were not associated with genetic differences of the DRD4 polymorphisms of the children or the mothers.

Analyses of gender differences did not show significant effects for CCQ personality measures neither for self-regulation nor for dysregulation. However, boys as compared to girls scored significantly higher on oppositional-aggression (*t*_(97)_ = 2.2, *p* = 0.023) and significantly lower on social competence (*t*_(97)_ = −2.9, *p* = 0.005). Therefore, gender was included as a covariate for the analyses regarding problem behavior.

### Concurrent and Longitudinal Correlations Between Personality Measures and Problem Behavior Measures

Next, we examined the concurrent convergence between the CCQ self-regulation self personality characteristics and the CCQ dysregulation variables for each age group. The correlations between the CCQ-personality variables separately for age six and age 12 (see Table 1) indicate the expected negative association between ego-resiliency and ego-undercontrol, CCQ aggressiveness, as well as CCQ anxiety both at age six and 12. Moreover ego-undercontrol and CCQ aggressiveness were positively associated whereas ego-undercontrol and CCQ anxiety did not correlate significantly at both age six and 12. The correlation between ego-undercontrol and CCQ aggressiveness was significantly higher than the correlation between ego-undercontrol and CCQ anxiety aggressiveness at age six (*z* = 9.56, *p* < 0.001) and at age 12 (*z* = 9.82, *p* < 0.001). CCQ aggressiveness and CCQ anxiety were not significantly associated.

**Table 1:** Correlations between CCQ personality variables at ages six and 12.

	Ego-resiliency	Ego-undercontrol	Aggressiveness	Anxiety
Ego-resiliency	-	−0.47***	−0.60***	−0.60***
Ego-undercontrol	−0.47***	-	0.84***	−0.26**
Aggressiveness	−0.58***	0.88***	0.	−0.15
Anxiety	−0.69***	−0.16	−0.09	-

Note. CCQ, California Child Q-sort; above the diagonal correlations for the 6-year assessment (*N* = 97); below the diagonal correlations for the 12-year assessment (*N* = 95). Note. **p ≤ 0.01, ***p < 0.001.

Next, we examined the concurrent associations between CCQ personality measures at age six and problem behavior scales at age six (see Table 2). Ego-resiliency was significantly positively associated with social competence and negatively with all forms of problem behavior, while high ego-undercontrol and CCQ aggressiveness were significantly related to low social competence, high oppositional aggressiveness, and inattentiveness/hyperactivity in the problem questionnaire. In addition, CCQ anxiety was significantly positively associated with affective problems in the problem behavior questionnaire.

**Table 2:** Concurrent correlations between children’s CCQ personality variables and problem behavior at age six.

CCQ	Social competence	Oppositional aggression	Inattentive-hyperactivity	Affective problems
Ego-resiliency	0.51***	−0.46***	−0.57***	−0.49***
Ego-undercontrol	−0.46***	0.61***	0.53***	−0.09
Aggressiveness	−0.57***	0.70***	0.42***	0.09
Anxiety	−0.08	−0.14	0.15	0.61***

Note. CCQ, California Child Q-sort (*N* = 97). Note. ***p < 0.001.

An analysis of the stability of the CCQ personality variables over time showed significant and moderately high differential stability over six years (see Table 3).

**Table 3:** Longitudinal correlations among CCQ-personality variables at ages six and twelve.

Age 12				
Age 6	Ego-resiliency	Ego-undercontrol	Aggressiveness	Anxiety
Ego-resiliency	0.37***	0.02	−0.10	−0.28**
Ego-undercontrol	−0.24*	0.50***	0.46***	−0.08
Aggressiveness	−0.21*	0.31**	0.43***	−0.10
Anxiety	−0.16	−0.40***	−0.32**	0.44***

Note. CCQ, California Child Q-sort (*N* = 89). Note. *p < 0.05, **p ≤ 0.01, ***p < 0.001.

### G x E Effects on Self-Regulation: Ego-Resiliency and Ego-Undercontrol

The DRD4 × maternal regulation × age tree-way MANOVA for ego-resiliency and ego-undercontrol revealed a main effect for maternal regulation (*F*_(2,82)_ = 3.47, *p* = 0.036, *η*^2^ = 0.078), a two-way interaction between maternal regulation and DRD4 (*F*_(2,82)_ = 3.89, *p* = 0.024, *η*^2^ = 0.087) and a three-way interaction between DRD4, maternal regulation, and age (*F*_(2,82)_ = 4.73, *p* = 0.011, *η*^2^ = 0.103).

**Figure 1:**
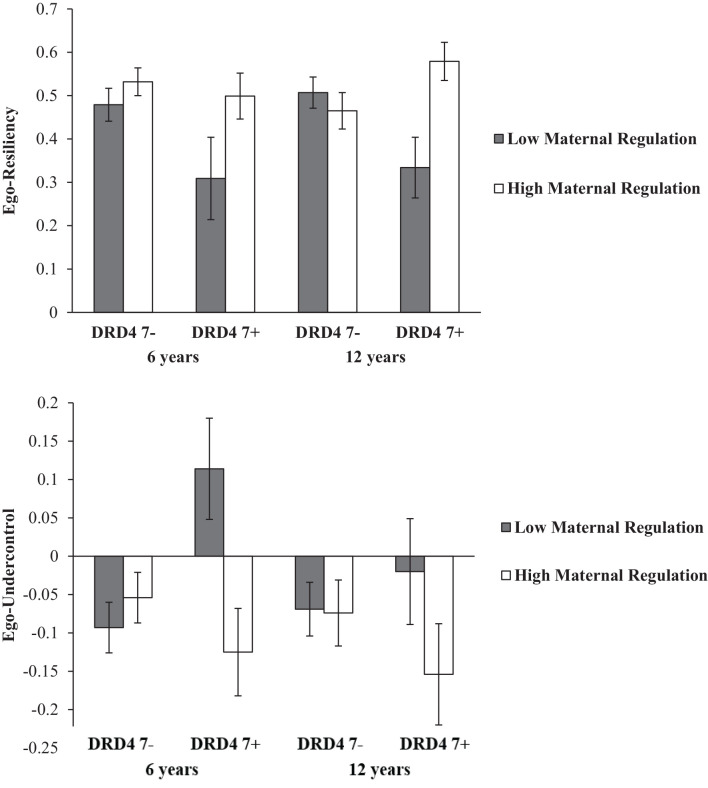
Children’s Means (and SE) in ego-resiliency and ego-undercontrol according to DRD4 polymorphism and maternalregulation.

Univariate analyses for ego-resiliency resulted in a significant main effect for maternal regulation (*F*_(1,83)_ = 5.76, *p* = 0.019, *η*^2^ = 0.065) and a significant interaction between maternal regulation and DRD4 (*F*_(1,83)_ = 5.52, *p* = 0.021, *η*^2^ = 0.062). Separate maternal regulation × age MANOVAs for the two DRD4 groups did not show significant effects for the DRD4 7- group, but a significant main effect for maternal regulation in children with DRD4 7+ (*F*_(1,20)_ = 8.6, *p* = 0.008, *η*^2^ = 0.301) indicating lower ego-resiliency in children of mothers with low emotional regulation in the DRD4 7+ group (see Figure 1).

Similarly, separate DRD4 × age MANOVAs for the two maternal regulation groups did not show effects for the group with high maternal regulation quality, but a main effect of DRD4 for children of the low maternal regulation group (*F*_(1,41)_ = 5.7, *p* = 0.021, *η*^2^ = 0.123), indicating lower ego-resiliency scores for DRD4 7+ children than DRD4 7- children in this group. Thus, as can be seen from Figure 1, both at six and at 12 years, low ego-resiliency mean scores were found for children with DRD4 7+, when they had experienced low maternal regulation, while the other three groups showed comparably high levels of ego-resiliency.

Univariate MANOVAs for ego-undercontrol also revealed an interaction between maternal regulation and DRD4 (*F*_(1,83)_ = 4.96, *p* = 0.029, *η*^2^ = 0.056). Separate maternal regulation × age MANOVAs for the two DRD4 groups showed a main effect for maternal regulation quality in children with DRD4 7+ (*F*_(1,20)_ = 6.05, *p* = 0.032, *η*^2^ = 0.232), while separate DRD4 × age MANOVAs for the two maternal regulation groups resulted in a main effect of DRD4 for the group of children with low maternal regulation (*F*_(1,41)_ = 4.26, *p* = 0.045, *η*^2^ = 0.094). As can be seen from Figure 1, children with DRD4 7+ who experienced low maternal regulation show more ego-undercontrol (at 6 years and at 12 years) than the other three groups exhibiting a moderate amount of ego-undercontrol.

Next, we considered effects of maternal responsiveness. The DRD4 × maternal responsiveness × age three-way MANOVA for ego-resiliency and ego-undercontrol revealed a significant three-way interaction between DRD4, maternal responsiveness, and age (*F*_(2,82)_ = 4.12, *p* = 0.020, *η*^2^ = 0.091). Univariate MANOVAs did not show a corresponding effect for ego-undercontrol but showed a significant three-way interaction for ego-resiliency (*F*_(1,83)_ = 8.34, *p* = 0.005, *η*^2^ = 0.091). Separate follow-up ANOVAs revealed a significant interaction between DRD4 and responsiveness at age 12 (*F*_(1,83)_ = 6.83, *p* = 0.011). According to *post-hoc* t-tests, children with less responsive mothers in the DRD4 7+ group at 12 years had lower ego-resiliency scores (*M* = 0.32) than the DRD4 7- group (*M* = 0.51), *T*_(41)_ = 2.36, *p* = 0.02).

### G x E Effects on Dysregulation: Aggressiveness and Anxiety

The DRD4 × maternal regulation × age three-way MANOVA for CCQ aggressiveness and CCQ anxiety revealed significant main effects for maternal regulation (*F*_(2,82)_ = 3.18, *p* = 0.050, *η*^2^ = 0.071) and age (*F*_(2,82)_ = 6.91, *p* = 0.002, *η*^2^ = 0.144), a marginal main effect for DRD4 (*F*_(2,82)_ = 3.05, *p* = 0.053, *η*^2^ = 0.069), and a significant three-way interaction between DRD4, maternal regulation and age (*F*_(2,82)_ = 5.19, *p* = 0.008, *η*^2^ = 0.075). Univariate analyses for CCQ aggressiveness resulted in a significant main effect for maternal regulation (*F*_(1,83)_ = 4.46, *p* = 0.038, *η*^2^ = 0.051) and a significant interaction between DRD4 and maternal regulation (*F*_(1,83)_ = 5.73, *p* = 0.019, *η*^2^ = 0.065), while for CCQ anxiety there were significant main effects for age (*F*_(1,83)_ = 9.94, *p* = 0.002, *η*^2^ = 0.107) and DRD4 (*F*_(1,83)_ = 5.36, *p* = 0.023, *η*^2^ = 0.061), and a significant three-way-interaction between DRD4, maternal regulation, and age (*F*_(1,83)_ = 6.41, *p* = 0.013, *η*^2^ = 0.072).

**Figure 2:**
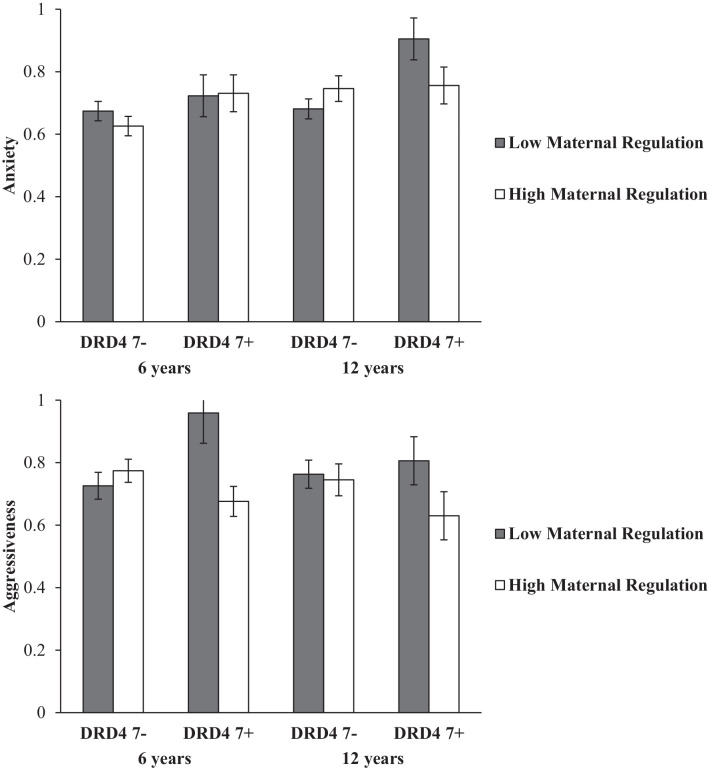
Children’s Means (and SE) in anxiety and aggressiveness according to DRD4 polymorphism and maternal regulation (Scores are lineary transformed by adding 1 to improve readability).

Regarding aggressiveness, separate maternal regulation × age MANOVAs for the two DRD4 did not show differences in CCQ aggressiveness depending on maternal regulation for the DRD4 7- group, but indicated significantly higher aggressiveness scores for children having experienced low as compared to high maternal regulation in the DRD4 7+ group (*F*_(1,20)_ = 7.68, *p* = 0.012, *η*^2^ = 0.277). Separate DRD4 × age MANOVAs for the two maternal regulation groups did not show significant effects. Thus, as can be seen in Figure 2, the effect of maternal regulation on CCQ aggressiveness was only found in the DRD4 7+ group. Both at 6 and at 12 years, CCQ aggressiveness of children with DRD4 7+ and low maternal regulation quality was higher in comparison to their counterparts with high quality of maternal regulation, while no differences regarding maternal regulation quality were found in DRD4 7- children.

For CCQ anxiety, separate follow-up ANOVAs for the two age periods revealed a significant main effect for DRD4 (*F*_(1,83)_ = 5.16, *p* = 0.026) and a significant interaction between DRD4 and maternal regulation quality (*F*_(1,83)_ = 4.13, *p* = 0.045) at age 12 but not at age six. *Post-hoc* t-tests revealed that at age 12 among the children of mothers with low maternal regulation quality the DRD4 7+ group had higher CCQ anxiety scores than the DRD4 7- group (*T*_(41)_ = 3.35, *p* = 0.002, see Figure 2).

The DRD4 × maternal responsiveness × age three-way MANOVA for CCQ aggressiveness and CCQ anxiety revealed a main effect for age (*F*_(2,82)_ = 6.29, *p* = 0.003, *η*^2^ = 0.133) and a three-way interaction between DRD4, maternal regulation, and age (*F*_(2,82)_ = 3.31, *p* = 0.041, *η*^2^ = 0.075). Univariate analyses did not show effects for CCQ aggressiveness. However, for CCQ anxiety, there were significant main effects for age (*F*_(1,83)_ = 9.83, *p* = 0.002, *η*^2^ = 0.106) and DRD4 (*F*_(1,83)_ = 5.28, *p* = 0.024, *η*^2^ = 0.060) and a significant three-way-interaction between DRD4, maternal regulation, and age (*F*_(1,83)_ = 5.08, *p* = 0.027, *η*^2^ = 0.056). Separate follow-up ANOVAs for the two age periods only revealed a significant main effect for DRD4 (*F*_(1,83)_ = 4.98, *p* = 0.028) at age 12 but not at age six, indicating that DRD4 7+ children had higher CCQ anxiety scores (*M* = 0.83) than DRD4 7- children (*M* = 0.76) at age 12.

### G x E Effects on Behavior Problems in Middle Childhood

**Figure 3:**
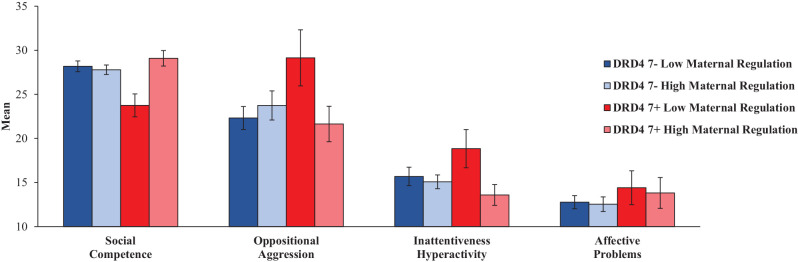
Children’s Means (and SE) in social competence, oppositional-aggression, inattentiveness/hyperactivity, and affective problems according to DRD4 polymorphism and maternal regulation.

The DRD4 × maternal regulation MANOVA (with gender as a covariate) for the four scales of the problem behavior questionnaire revealed a significant multivariate interaction effect between DRD4 and maternal regulation quality (*F*_(4,83)_ = 2.71, *p* = 0.036, *η*^2^ = 0.117). Univariate follow-up analyses showed a significant interaction effect for social competence (*F*_(1,85)_ = 9.97, *p* = 0.002, *η*^2^ = 0.105) and a statistical trend of the interaction for inattentiveness/hyperactivity (*F*_(1,85)_ = 3.01, *p* = 0.087, *η*^2^ = 0.034), and oppositional-aggression (*F*_(1,85)_ = 3.72, *p* = 0.057, *η*^2^ = 0.042). *Post hoc* comparisons of the problem behaviors of children of mothers with low or high maternal regulation quality, conducted separately for the two DRD4 groups and controlling for gender effects revealed a significant effect of maternal regulation on social competence (*F*_(1,19)_ = 6.87, *p* = 0.017) indicating lower social competence in children of mothers with maternal low regulation in the DRD4 7+ group. Comparing the two DRD4 groups separately, an analysis for the two maternal regulation groups revealed a significant DRD4 7+ effect on oppositional-aggression (*F*_(1,43)_ = 4.49, *p* = 0.040) and social competence (*F*_(1,43)_ = 9.59, *p* = 0.003) in the low maternal regulation quality group, indicating more oppositional aggression and less social competence in DRD4 7+ children experiencing low maternal regulation quality already in infancy (see Figure 3). In addition, the children with DRD4 7+ and low maternal regulation compared to the group with high maternal regulation quality showed significantly lower social competence scores (*F*_(1,20)_ = 11.2, *p* = 0.003), significantly higher scores in inattentiveness/hyperactivity (*F*_(1,20)_ = 4.5, *p* = 0.047), and, as a statistical trend, a higher mean score in oppositional-aggression (*F*_(1,20)_ = 3.9, *p* = 0.06).

In contrast, the DRD4 × maternal responsiveness two-way MANOVA for the four scales of the problem behavior questionnaire did not reveal significant main or interaction effects.

## Discussion

The main objectives of this study were to investigate the effects of early maternal caregiving and molecular genetic polymorphisms of the dopamine receptor gene (DRD4) on self-regulation in middle childhood and adolescence. We examined effects on personality characteristics assessing self-regulation and dysregulation in middle childhood and adolescence, and in addition problem behavior in middle childhood. As the participating families had not experienced any peri-natal or post-natal complications early risk factors influencing later self-regulation at the time of first assessment will not explain differences in self-regulation (Feldman, 2009; Bersted and DiLalla, 2016).

### No Genetic Influences on Maternal Sensitivity

We first examined whether differences in maternal sensitivity could be explained by evocative processes based on the children’s genetic dispositions or influenced by maternal genetic variations (Mills-Koonce et al., 2007; Reiss and Leve, 2007), suggesting a gene-environment-correlation (Rutter, 2006). We did not find any effects of mothers’ or children’s DRD4 polymorphisms on maternal sensitivity variables. Thus, the effects of early maternal regulation on the children’s later personality in this study cannot be explained by gene-environment-correlation (Rutter, 2006), neither as an epi-phenomenon of the mothers’ nor the children’s DRD4 polymorphisms. This is in line with other studies examining the effect of genetic dopaminergic polymorphisms reporting no empirical evidence for a gene-environment correlation of supportive parenting and DRD4 polymorphisms (Cho et al., 2016), maternal sensitivity and maternal DRD1 or DRD2 A1 polymorphisms (Mills-Koonce et al., 2007; Mileva-Seitz et al., 2012). Maternal sensitivity as the appropriate adjustment and regulation of one’s own parenting behaviors to children’s signals might be less affected by typical dopaminergic functions (i.e., regulation of attention, inhibition, or reaction to reward). However, the frequency of single, specific parenting behaviors (e.g., orientation to child) might well be influenced by genetic differences of the dopaminergic system (Mileva-Seitz et al., 2012) and other aspects of parenting may show systematic variations depending on polygenetic scores (Wertz et al., 2019). However, other factors explaining differences in maternal sensitivity may play a role (e.g., mother’s attachment history or maternal depression; Pederson et al., 1998; Grossmann et al., 2008; Bernard et al., 2018) or other genetic differences (Mileva-Seitz et al., 2011). In general, the results offer no empirical evidence that the two dimensions of maternal sensitivity assessed in this study, maternal regulation quality and maternal responsiveness, are a result of specific differences in mothers’ DRD4 7+ vs. 7-polymorphisms or an evoked reactive reaction based on DRD4 variation of the child. Thus, we interpret maternal effective regulation and responsiveness as independent individual psychological factors influencing later child development.

### Ego-Resiliency and Ego-Undercontrol: Continuity and Associations With Dysregulation and Maladjustment

The study shows a moderate stability of ego-resiliency and ego-control in this sample which is comparable to the original study by Block and Block (2006) and a Swedish longitudinal study (Chuang et al., 2006; Syed et al., 2020) and is close to the mean stability of personality traits at that age as reported in a meta-analysis (Roberts and DelVecchio, 2000). The moderate differential stability suggests some changes in rank order of all personality characteristics. Thus, we still find differential developmental trajectories in personality development with both growth and decline in self-regulation and dysregulation between middle childhood and early adolescence which may also be due to other reasons like differences in the onset of puberty within the sample (Block and Block, 2006).

Moreover, the study shows that ego-resiliency in middle childhood and in adolescence as a sign of flexible self-regulation is clearly associated with lower scores in aggressiveness and anxiety at age six and age twelve (i.e., personality traits assessing dysregulation) and also with fewer problem behaviors and more social competence in middle childhood. These findings corroborate the relevance of ego-resiliency as a fundamental dimension of effective self-regulation and adjustment (Block and Block, 2006; Taylor et al., 2014; Syed et al., 2020). Ego-resiliency is a marker of effective and situation-appropriate adjustment of self-control and goes along with both low internalizing and low externalizing symptoms from early childhood to adolescence (Scholte et al., 2005; Martel and Nigg, 2006; Hofer et al., 2010; Meier and Zimmermann, 2018; Zimmermann et al., 2022).

Interestingly, ego-undercontrol shows a differential pattern of associations with concurrent signs of externalizing (i.e., aggressiveness, inattention-hyperactivity) and internalizing (i.e., anxiety). Ego-undercontrol is positively associated with concurrent CCQ aggressiveness at age six and age twelve. Moreover, at age six it is also significantly correlated with concurrent oppositional aggression and inattentive-hyperactivity. Thus, children who immediately express or act according to their current needs, emotions, or goals, regardless of context, and who demonstrate poor delay of gratification show more externalizing symptoms and are rated low in social competence. In contrast, ego-undercontrol is negatively associated with CCQ anxiety at age six and negatively but not significantly at age twelve. The significantly different correlations of ego-undercontrol with aggressiveness compared to anxiety are in line with other research (Krueger et al., 1996) and can already be found in early childhood (Zimmermann et al., 2022). It characterizes the specific theoretical concept of ego-control as a continuum from undercontrol to overcontrol (Funder and Block, 1989; Block, 2002; Block and Block, 2006). Thus, anxiety, assessed as a personality trait here, may not only be a sign of hyperactivating emotion regulation (i.e., low self-regulation) but also a sign of constant overcontrol. Recent research supports Block’s theoretical perspective by showing a U-shaped association between self-control and mental health problems (Hassan and Schmidt, 2021) where children with low levels of self-control and also children with high levels of self-control show increased internalizing and externalizing problems. Self-regulation and self-control are sometimes conceptually confounded in research but may have different implications for adjustment or clinical symptoms (Nigg, 2017).

### Gene-Environment Effects

In the present study, two main results of the gene x environment effects on personality development are of special interest. First, there are main effects for early maternal caregiving and a moderation of the genetic disposition associated with the long (7+) variant of the DRD4 polymorphism by early maternal caregiving. This is the case for all three proposed levels of self-regulation capacities, personality characteristics assessing *self-regulation*, personality characteristics assessing *dysregulation*, and also the third level of enduring problems with self-regulation for most *mental health problem* domains and social competence. Second, specifically effective maternal regulation quality but not maternal responsiveness is a moderator of DRD4 polymorphism in these gene-environment interactions.

The general pattern of results shows that effective early maternal regulation in infancy predicts variation in self-regulation (specifically ego-resiliency) and in dysregulation (specifically aggressiveness). The significant interaction effects found for personality differences in self-regulation and dysregulation and also for problem behavior (i.e., all three levels of regulatory capacity) suggest that, for the group of children with the DRD4 7+ variant, effective maternal regulation experienced already in infancy can compensate for the negative genetic disposition associated with the DRD4 7+ variant on flexible self-regulation as a personality trait. This is in concordance with other studies on gene × environment interaction for DRD4 polymorphisms (Bakermans-Kranenburg and van IJzendoorn, 2006; Sheese et al., 2007; Bakermans-Kranenburg and van IJzendoorn, 2011; Martel et al., 2011) and also for DRD2 A1 polymorphisms (Mills-Koonce et al., 2007; Waldman, 2007). However, some studies assessing other parenting variables and other aspects of children’s self-regulation do not report similar results. This can be seen in the study by Sheese et al. (2007), where parenting quality was assessed with an aggregated score of supportive presence, autonomy support, cognitive stimulation, and low hostility. Here, low (aggregate) parenting quality and DRD4 7+ disposition resulted in a low level of sensation seeking (defined as impulsive, cheerful activity) but the study showed no gene-environment interaction for effortful control (an aggregate of attention regulation and inhibitory control) as a sign of self-control. We conclude that not all parenting variables may function as moderators of genetic predispositions for all self-control variables. As a consequence, we need to differentiate specific aspects of parenting or parental sensitivity that may help infants to develop enduring self-regulation from other domains of parental sensitivity that do not have the same effect. Many studies on gene-environment-interaction only use a global or aggregated score of sensitivity, parenting, or caregiving, often only because the single caregiving variables are correlated. However, correlations between different measures of sensitivity often are only modest or rather modest (Lohaus et al., 2004; Bohr et al., 2018). Future studies should have a closer look at the differential functions of caregiving variables and contexts for the development of attachment and self-regulation in gene-environment studies (Golds et al., 2020; Picardi et al., 2020).

Direct effects of the DRD4 polymorphisms on children’s personality only appeared for CCQ anxiety, specifically at age twelve. Thus, although the personality variables chosen in this study are all related to differences in functions of the dopamine system (inhibition and regulation of impulses, emotions, and behavior) a direct main effect of DRD4 does not explain the developmental pathway of personality development for all of these variables. As we do not have data on the mental health problems or problem behavior at age 12 we cannot examine whether the results on affective problems at age six also would replicate at age 12. This warrants further longitudinal assessments.

In sum, the results suggest that children with the DRD4 7+ variant and without early effectively regulating parenting already in infancy will develop less effective self-regulatory abilities. For these children it is highly relevant to experience early effective external regulation to learn the ability to flexibly control and modify their behavioral and emotional reactions in early caregiver-child interaction. The results seem to support the notion of children’s differences in susceptibility for environmental conditions, specifically effective parenting (Belsky and Pluess, 2013; Belsky and van IJzendoorn, 2017). Thus, while the personality development of children with the 7+ variant of the DRD4 polymorphism depends on specific qualities of maternal caregiving (i.e., early effective regulation) children with the 7- variant seem not to be affected by differences in such early maternal caregiving. This is in line with other studies on gene × environment effects (Caspi et al., 2002). The role of the dopaminergic system in the development of self-regulation in childhood may be especially seen rather early in development (Posner et al., 2007). According to Posner and Rothbart (2022), dopamine is a specific modulator of the executive function domain of the attention network, where DRD4 polymorphisms already have an early functional impact during development. The dopamine pathways are involved in motor control and planning, cognitive processes, and reward processing and therefore influence the regulation of attention but also of emotions and motivation (Nieoullon and Coquerel, 2003). Animal studies suggest, that specifically the flexibility and efficiency of self-regulation are impaired. Some research suggests that the *DRD4* 7+ variant is associated with reduced dopaminergic signaling leading to reduced learning from external stimuli or caregiving (Tripp and Wickens, 2008). Therefore, by reducing the child’s emotional arousal, effective maternal regulation may be more effective in fostering children’s early self-regulation than fast maternal responding.

Besides the children’s susceptibility, effective maternal regulation in infancy was more influential than maternal responsiveness in this study. While effective maternal regulation showed a repeated and systematic effect on different personality measures at different ages maternal responsiveness only showed an effect on ego-resiliency at age 12. This suggests that the caregiver’s ability to effectively regulate the infant’s negative emotions as well as to assist the child effectively during exploration seems to help the child to develop effective and adaptive patterns of self-regulation (Thompson, 1993; Spangler et al., 1994; Grossmann et al., 2008; Bernier et al., 2010). In contrast, other aspects of sensitivity like vigilant and fast reactivity to children’s signals may not always be a sign of supportive caregiving if combined with problems in effectively regulating the child’s emotional states or if combined with actively dysregulating the child by being intrusive. Thus, prompt maternal reactions alone do not necessarily support children during emotion regulation processes and children consequently do not learn effective self-regulation even when parents are highly vigilant to their emotional expressions. Similarly, Lohaus et al. (2004) reported that reactions appropriate to infants’ needs, but not signal perception of infants’ expression was predictive of a low rate of infant crying, a sign of effective self-regulation (Lohaus et al., 2004). Moreover, parental sensitivity and parental intrusiveness may differentially influence the intercept and slope of self-control development (Geeraerts et al., 2021). For other genetic predispositions and also for other developmental outcomes besides self-regulation, specific aspects of sensitivity or caregiving by both mothers and fathers or attachment security may well play an important role (Spangler et al., 2009; Zimmermann et al., 2009; Davies et al., 2015; Zimmermann and Spangler, 2016; Baptista et al., 2017; Neppl et al., 2020; Lee et al., 2021).

The effect of early maternal regulation for the development of self-regulation and for problem behavior in childhood and early adolescence suggests the importance of early sensitive experiences on personality development. Following the model of stage-salient issues (Sroufe, 1989), effective regulation of the infant’s negative emotions is the earliest stage-salient issue with potential effects on later stage-salient issues influencing subsequent personality development and adjustment (Cicchetti and Toth, 1992; Toth and Manly, 2019). Studies on long-term effects of early sensitivity provide support for this assumption (Beckwith et al., 1992; Grossmann et al., 2002). However, given the gene × environment interaction effects of DRD4 and maternal regulation found in this study, the long-term effects of early maternal regulation might specifically help children with a genetic disposition for problems to adjust their behavioral expressions of own emotions, needs, or goals to the affordances of the current situation, obvious as behavioral dysregulation.

For problem behavior, we found a main effect for maternal regulation quality and a similar interaction effect between maternal regulation quality and DRD4 polymorphism. Again, early regulation of the children’s emotional and exploratory needs helps children with the 7+ variant of the DRD4 polymorphism to reach a comparably high level of social competence and a comparably low level of oppositional aggression and attention-hyperactivity problems similar to the children with the DRD4 7- variant. A similar compensatory effect of maternal sensitivity for carriers of the DRD4 7+ variant on externalizing behavior in preschool children was reported by Bakermans-Kranenburg and van IJzendoorn (2006). Many studies have shown that the DRD4 7+ polymorphism increases the risk for the development of ADHD (Thapar et al., 2007b; Bonvicini et al., 2020) and contributes to its stability over time (El-Faddagh et al., 2004). However, the base rate of children diagnosed with ADHD showing the DRD4 7+ polymorphism is seldom higher than 50%. The present study suggests that even a biological predisposition for attention-hyperactivity problems and oppositional aggression might well be compensated quite early in life, leading to at least an average ability for self-regulation that can be helpful for adjustment and adaptive self-regulation at a non-clinical level. Thus, social influences on the development of attention problems and hyperactivity should be considered more closely as a second developmental pathway showing equifinality (Carlson et al., 1995; Pauli-Pott et al., 2018) when maternal regulation is low and given the DRD4 7 repeat polymorphism. In future research, the timing of caregiving effects on self-regulation needs to be explored in more detail (Weeland et al., 2015).

The present study has some limitations that need to be considered. The self-regulation variables and the dysregulation variables were assessed with the same instrument, potentially increasing their shared variability. The effect sizes for the main effects for maternal regulation and the interaction effects of DRD4 polymorphisms and maternal caregiving are relatively small and also the number of children with the 7+ polymorphism, although comparable to other studies published in the field. In addition, the effects of cumulative environmental risks (Caspi et al., 2003; El-Faddagh et al., 2004; Reiner and Spangler, 2011) or the interaction with other genetic variations (Schmidt et al., 2007; Wertz et al., 2019) could offer even more insights in developmental processes. Moreover, a GWAS approach might be more representative of the complex interplay of genetic variations on personality development and adjustment. However, tandem repeats seem to have more impact on the development of self-regulation or mental health problems than expected earlier (Xiao et al., 2021). Thus, replications are required as well as research designs with additional assessment waves to identify developmental processes of self-regulation and dysregulation and to examine the relevance of early compared to later caregiving or other social experiences more closely.

## Conclusion

We conclude that especially early effective maternal regulation of infants’ distress can help children with the DRD4 7+ variant to develop adaptive self-regulation in middle childhood and adolescence. We suggest that research on gene × environment effects not only should seek for all possible genetic variations influencing self-regulation but simultaneously also should more closely examine the environment and the existing variations in caregiving behaviors to better understand developmental processes in the interplay of social and genetic influences on personality development and (mal-) adjustment.

## Data Availability Statement

The original contributions presented in the study are included in the article, further inquiries can be directed to the corresponding author.

## Ethics Statement

The studies involving human participants were reviewed and approved by Ethics Committee of the German Psychological Association. Written informed consent to participate in this study was provided by the participants’ legal guardian/next of kin.

## Author Contributions

Both authors contributed equally to the study design, the statistical analysis, and the writing and editing of the manuscript. All authors contributed to the article and approved the submitted version.

## Conflict of Interest

The authors declare that the research was conducted in the absence of any commercial or financial relationships that could be construed as a potential conflict of interest.

## Publisher’s Note

All claims expressed in this article are solely those of the authors and do not necessarily represent those of their affiliated organizations, or those of the publisher, the editors and the reviewers. Any product that may be evaluated in this article, or claim that may be made by its manufacturer, is not guaranteed or endorsed by the publisher.
